# Opportunities and considerations for the design of decentralized delivery of antiretroviral therapy for female sex workers living with HIV in South Africa

**DOI:** 10.1186/s12913-022-08506-0

**Published:** 2022-09-16

**Authors:** Carly A. Comins, Vijayanand Guddera, Lauren E. Parmley, Katherine Young, Mfezi Mcingana, Ntambue Mulumba, Sharmistha Mishra, Deliwe R. Phetlhu, Harry Hausler, Sheree Schwartz, Stefan Baral

**Affiliations:** 1 Department of Epidemiology, Johns Hopkins Bloomberg School of Public Health, 615 N Wolfe St, Baltimore, MD 21205 USA; 2 TB HIV Care, Durban, South Africa; 3 United States Agency for International Development, Pretoria, South Africa; 4 TB HIV Care, Cape Town, South Africa; 5 Li Ka Shing Knowledge Institute, St. Michael’s Hospital, University of Toronto, Toronto, Canada; 6 Division of Infectious Diseases, Department of Medicine, University of Toronto, Toronto, ON Canada; 7 School of Health Care Sciences, Sefako Makgatho Health Sciences University, Ga-Rankuwa, South Africa; 8 Department of Epidemiology, Johns Hopkins School of Public Health, Baltimore, USA

**Keywords:** HIV, Female sex workers, South Africa, Antiretroviral, Differentiated care, Differentiated service delivery

## Abstract

**Background:**

In South Africa, 60% of female sex workers (FSW) are living with HIV, many of whom experience structural and individual barriers to antiretroviral therapy (ART) initiation and adherence. Community-based decentralized treatment provision (DTP) may mitigate these barriers. To characterize optimal implementation strategies, we explored preferences for DTP among FSW living with HIV in Durban, South Africa.

**Methods:**

Thirty-nine semi-structured in-depth interviews were conducted with FSW living with HIV (*n* = 24), and key informants (*n* = 15) including HIV program implementers, security personnel, and brothel managers. Participants were recruited using maximum variation and snowball sampling. Interviews were conducted in English or isiZulu between September–November 2017 and analyzed using grounded theory in Atlas.ti 8.

**Results:**

DTP was described as an intervention that could address barriers to ART adherence and retention, minimizing transport costs, time and wage loss from clinic visits, and act as a safety net to address FSW mobility and clinic access challenges. Respondents highlighted contextual considerations for DTP and suggested that DTP should be venue-based, scheduled during less busy times and days, and integrate comprehensive health services including psychological, reproductive, and non-communicable disease services. ART packaging and storage were important for community-based delivery, and participants suggested DTP should be implemented by sex work sensitized staff with discrete uniform and vehicle branding.

**Conclusions:**

Incorporating FSW preferences may support implementation optimization and requires balancing of tensions between preferences and feasibility. These data suggest the potential utility of DTP for FSW as a strategy to address those most marginalized from current ART programs in South Africa.

## Background

As antiretroviral therapy (ART) programs expand, global funding for the HIV response is plateauing or even decreasing [1, 2], and donors and national health systems are re-evaluating how ART care is delivered. The World Health Organization (WHO) recommends adaptive or differentiated approaches to HIV care to maximize reach and effectiveness [3]. Differentiated service delivery (DSD), a patient-centered approach to HIV care, aims to more effectively target care and reduce the burden on the health system [4, 5], and can be facility- or community-based [6–8]. Globally, the focus of DSD to-date has largely been ART provision for clinically stable (virally suppressed) adult chronic care patients [4, 5]. Through recommendations from key stakeholders [3, 9–11], DSD has increasingly expanded to more vulnerable, but still clinically stable populations including children, adolescents, pregnant and breastfeeding women, and key populations (KPs) including female sex workers (FSW) [9–11]. DSD builds on existing global standards of HIV care [12–15], and can aid in addressing structural barriers to ART access, adherence, and retention through population-specific provision of tailored care [16, 17]. However, scalable DSD strategies must be adaptable, acceptable, and cost-effective to specific populations [10, 18].

In 2021, there were an estimated 7.8 million people living with HIV in South Africa, of which 7.26 million knew their HIV status, 5.6 million were on ART, and 5.1 million had suppressed viral loads [19]. Despite progress in ART scale-up, treatment roll-out remains below UNAIDS 90–90-90 targets [18, 19] and HIV risk and burden are not evenly distributed. HIV prevalence and treatment coverage among FSW in South Africa are estimated to be 62% and 39%, respectively [2, 19, 20], and the prevalence of viral load suppression, where available, are generally suboptimal [21, 22].

Decentralized treatment provision (DTP), a DSD strategy that includes community-based provision of ART, may address barriers to ART linkage, uptake, and retention among FSW [23–27] and leverages priorities for community-based, nurse-led ART care and treatment distribution [28]. DTP at select pick-up points (i.e., designated pharmacy queues, churches, and schools) has been implemented as part of the South African National Department of Health response since 2014 through the Central Chronic Medicine Dispensing and Distribution (CCMDD) program, but it has been limited to virologically suppressed adults living with HIV and on treatment for a sustained period; the CCMDD does not address the unique needs of marginalized populations or those not virally suppressed [29, 30]. This is despite evidence across sub-Saharan Africa that community-based ART distribution for key populations (including but not limited to FSW) has been found to be as effective as facility-based care and resulted in similar HIV clinical outcomes [31].

In sum, most research on DSD predominately compares differentiated models to traditional treatment models, often failing to incorporate user preferences [32, 33]. Understanding and embedding preferences for FSW in the implementation of DTP will support the appropriateness, adoption, and acceptability of the intervention’s implementation [34, 35]. Further, assessing user preferences allows for DSD packages to be tailored to populations and aware of heterogeneity within populations [32]. This formative qualitative research aimed to characterize the opportunities and considerations for DTP implementation for FSW living with HIV in Durban, South Africa.

## Methods

Data were collected in Durban, South Africa in collaboration with TB HIV Care (THC), as part of the formative research for the Siyaphambili Study [36], from September–November 2017. The THC FSW program has been active in Durban since 2012, and includes mobile van-based HIV testing and prevention services for FSW, and a facility-based drop-in center staffed by nurses, peer educators, and counselors where ART is provided for free in line with national treatment guidelines [28, 37].

Twenty-four in-depth interviews (IDIs) and 15 key informant interviews were conducted between September and November 2017. IDIs were conducted with cisgender FSW who were 18 years or older, sold sex as their primary source of income in the last 12 months, self-reported living with HIV, and resided in Durban. To address FSW heterogeneity, IDI participants were recruited using maximum variation sampling to ensure variability across age, venue type, time of operation, treatment experience, and proximity from sex work venue to the THC drop-in center [38, 39]. Recruited by peers, FSW participants were identified at sex work venues and the THC drop-in center. Reimbursement of 100 ZAR (~ 7 USD) was provided to FSW participants.

Key informants (KIs) were recruited purposively and through snowball sampling [40] and included FSW program staff (manager, nurses, counselors, peers, and drivers) from government and non-governmental organizations, security (police and neighborhood watch personnel), and brothel managers. Eligibility criteria for KIs included stakeholders who were 18 years or older and had experience in HIV programming or policy for FSW or could speak to the needs of FSW.

Prior to data collection, written informed consent was obtained from participants. All interviews were conducted in a private location (i.e., THC drop-in center, THC mobile van, sex work venue, or other prespecified community location) by one qualitative interviewer trained and experienced in qualitative methods and human subjects’ research. Semi-structured interview guides focused on potential barriers and facilitators of DTP intervention through the mobile van to promote ART access and adherence for FSW living with HIV. Interviews lasted between 60–80 min, were conducted in isiZulu or English, and were audio-recorded. Participation was limited to one interview per person. Recordings were transcribed verbatim and translated into English by an external individual trained and experienced in transcription and translation of qualitative interviews.

Analysis occurred in a two-phased, cyclical process. After each interview, a memo was drafted by the interviewer to capture key themes and field notes [41]. Memos and emerging themes were discussed on a weekly basis by the study team; data collection and interpretation were iterative. A codebook was developed based on emergent themes and DSD strategies. Each transcript was coded by co-authors CAC and LP, discussed, and coding discrepancies resolved. Data were managed using Atlat.ti 8 and analyzed using a grounded theory approach [42]. Themes have been summarized in the text and illustrative quotes presented within the text and tables. Names included are pseudonyms provided by participantd. Additionally, DSD considerations were mapped across the building blocks of differentiated ART delivery for KPs [10]. Results represent themes that reached data saturation [43].

This study was approved by the Johns Hopkins Bloomberg School of Public Health Institutional Review Board (IRB No. 00007847), the University of Western Cape Biomedical Research Ethics Committee, and the KwaZulu-Natal Department of Health.

## Results

Among the 24 FSW participants, half reported current ART use (Table 1). More than half of FSW met or solicited clients at outdoor sex work venues (e.g. streets, parks) and 63% operated during the day. KIs were primarily female and over half were older than the age of 40 (Table 2).

**Table 1:** Demographic characteristics of female sex workers participating in in-depth interviews, Durban, South Africa (*n* = 24)

Characteristic	n (%)
**Age**	
18–25	4 (16.7%)
26–30	5 (20.8%)
31–35	9 (37.5%)
36–40	4 (16.7%)
41–50	2 (8.3%)
**ART-naïve**	
No	20 (83.3%)
Yes	4 (16.7%)
**Currently on ART**	
No	12 (50.0%)
Yes	12 (50.0%)
**Venue Type**	
Indoor^a^	9 (37.5%)
Outdoor ^b^	15 (62.5%)
**Primary time of work**	
Daytime	15 (62.5%)
Nighttime	6 (25.0%)
Both	3 (12.5%)
**Distance from sex work venue to drop in center**^c^	
Near	18 (75.0%)
Far	6 (25.0%)

*Abbreviations: ART* Antiretroviral therapy

^a^Indoor venues: hotels, home-based brothels, strip clubs with accommodations

^b^Outdoor venues: corners/streets/street corners, taxi rank, truck stops

^c^Far venues: defined as needing to take more than one taxi or bus to get to the THC drop-in center

**Table 2:** Demographic characteristics of key informants, Durban, South Africa (*n* = 15)

Characteristics	n (%)
**Sex**	
Male	6 (40.0%)
Female	9 (60.0%)
**Race**^a^	
Black African	7 (46.7%)
Coloured	4 (26.7%)
Indian	2 (13.3%)
White	2 (13.3%)
**Age (years)**	
26—30	3 (20.0%)
31—35	3 (20.0%)
36—40	1 (6.6%)
41—45	4 (26.7%)
46 +	4 (26.7%)
**Role**	
FSW program staff^b^	8 (53.4%)
Security (police/neighborhood watch)	2 (13.3%)
Professional nurse	4 (26.6%)
Brothel owner	1 (6.7%)

^a^Based on official race categories in South Africa

^b^Program staff included program manager, peer educators, counselor, driver from Department of Health and non-governmental organizations

### Opportunities for community-based decentralized treatment provision of ART

Most FSW expressed interest in receiving ART at sex work venues. Primary opportunities for DTP included saving time and money, creating a safety net to address barriers caused by FSW mobility and challenges accessing the clinic, and protecting confidentiality, as described in detail below. Illustrative quotes highlighting sub-themes are presented in Table 3.

**Table 3:** Opportunities for community-based decentralized treatment provision of ART

Opportunities	Illustrative Quotes
**Time is money…**	
… to reduce time away from work	*… taking treatment is not a problem. The issue is leaving work in order to collect treatment… My clients will also find me easily because I would be collecting my treatment nearby. I won’t lose any client and I won’t default. (Kim, Hotel-based FSW)* *Delivering ART is a good idea… Especially to where we work. Let’s put an example like your [clinic] date is there on Friday and Friday you know is a busy day [for sex work]. No, I’ll go on Monday. So at least when they come on Friday you just take your pills and put it in your room. (Lindiwe, Hotel-based FSW)*
…to remove cost associated with attending the clinic	*Sometimes you run out of money and find that it is your date to go to the clinic and your test is money because there is nothing you can do if there is no money. (Mpintshi, outdoor-based FSW)* *Leaving home to get to the clinic costs money. I also need to use money to go to work. I need to budget accordingly. Sometimes I end up not going to the clinic because I don’t have money to get there. But if medication is delivered to me at work, one trip is saved because they will find me there.* (*Stacey, home-based brothel FSW)*
**Safety net…**	
… to address mental incapacitation	*Even in your high states of mind, when you’re high if you see the clinic it will bring back memory, hey, I’m supposed to do this. I’m suppose to do that. (Dione, outdoor-based FSW)* *It will help me a lot because I’m a very forgetful person I always forget my dates…at least that is going to help me to remind me. (Melisa, outdoor-based FSW)*
…to address FSW mobility	*You can’t say like you haven’t seen [the mobile van] because it is everywhere. (Dione, outdoor-based FSW)* *…[DTP] can be of much help since we are unable to use local clinics because they want proof of residence and we don’t have since we live in these [temporary] houses… So it will help us because they [DTP implementors] will not want all these things that we don’t have. (Ayanda, outdoor-based FSW)*
**Confidentiality…**	
…to address unintended HIV status disclosure at clinic	*People are afraid to go to clinics because neighbors will judge them. Like myself, I decided to collect my treatment far away from my local clinic. If you use the mobile clinic then no one will know what you are doing there because there is only one queue to enter. (Brenda, outdoor-based FSW)*
… to protect confidentiality through service integration	*No I am not afraid [a client will see me accessing the mobile van] because he may not be sure. He will think that I went to the mobile just to ask about something else, maybe I am on my periods, maybe I’m pregnant or I have something else. (*Natasha, hotel-based FSW) *I do not care if the clients know, but I think they should ask me so that I can explain what is happening because it is a mobile clinic which does not mean it is for people living with HIV. There are many things the mobile clinics offer like, condoms, lubricators, pills and to cure STIs even if you had a condom burst with the client, they also help you with check-ups. It is important to explain to clients about the mobile because it also helps them, and it is important to know your status.* (Lungo, hotel-based FSW) *It is more discrete. When she comes to the mobile it is not like she’s coming for treatment because they [FSW] all come to the mobile when we come to a [sex work] site; whether you’re negative or positive. You could be coming for a retest, you could be coming for family planning, you could be coming for a whole lot of reasons. When they walk out with condoms they’re walking out with a plastic packet. (*Female, FSW program staff)

#### Time is money

All FSW participants expressed that DTP could address structural barriers to ART access and retention, specifically noting DTP would save FSW time and money. FSW emphasized that taking ART was not difficult, however, leaving sex work venues to collect treatment at the clinic posed challenges as it required time, money, and resulted in potential income loss (i.e. from clients, daily rent, or money paid to manager/pimp). FSW noted that DTP at sex work venues would address these challenges, enabling women to obtain treatment onsite, return to work quickly, and reduce time spent away from clients.

DTP was suggested to reduce time spent traveling to and at the clinic as well as reduce transportation costs. While a few FSW reported having to ‘hustle’ harder or budget appropriately to ensure they had money to visit the clinic during work hours, many reported not visiting clinics due to cost including opportunity cost. DTP at venues was also seen as an opportunity to replace long clinic queues and wait times, but also duration of time spent at the clinic, as FSW could obtain treatment, conduct necessary bloodwork, and receive routine services on the spot and near their place of work. A few women stated that DTP would benefit their overall health and wellbeing, noting that it would prevent treatment defaulting and enable women to save money (e.g. to buy food).

#### Safety net

DTP at sex work venues was also viewed as an opportunity to address barriers caused by mobility and act as a safety net for mobile FSW. Firstly, DTP would ensure follow-up and facilitate attendance of scheduled appointments for FSW lacking transport money. Some FSW responded that they were unable to access clinics because of work-related fatigue (e.g. working throughout the night) or due to effects of intoxication and/or drugs. These participants expressed that seeing the mobile van at their venue could not only enable access to ART but could remind them to take their treatment.

Additionally, DTP at sex work venues emerged as an opportunity to allow for and address mobility of FSW in South Africa. Specifically, KIs emphasized DTP the value of flexible implementation on DTP to allow for FSW movement across and between sex work venues to avoid police arrests or hassle. A few KIs also reported that ART delivery onsite could assist recently relocated FSW who face barriers to accessing care due to their unfamiliarity with local clinics, not having the clinic’s address, or lack of clinic cards required to access treatment.

#### Confidentiality

Protecting FSW’s confidentiality and HIV-positive status emerged as another major opportunity of DTP. Most participants identified fear of unintended HIV disclosure to boyfriends or known community members as a key barrier to accessing HIV treatment from government clinics. Participants feared community members learning their HIV statuses as a result of seeing them at the clinic and explained that receiving ART from the THC mobile van would be discrete and confidential as it offers a wide range of health services. KIs reiterated that the offering of additional services on the mobile van and at the point of distribution reduces the likelihood of inadvertent disclosure to other FSW or clients.

### Considerations for community-based DTP of ART

**Fig. 1:**
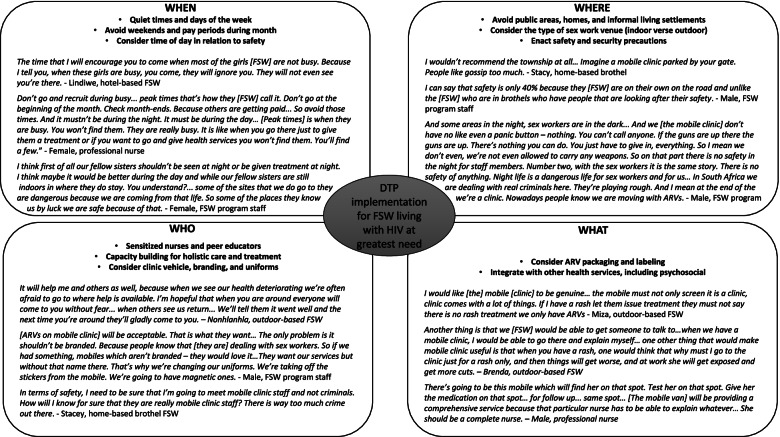
Participant preferences surrounding DTP implementation for FSW living with HIV at greatest need

The importance of co-designing DTP strategies with FSW and tailoring strategies to focus specifically on FSW strongly emerged. FSW and KI considerations are summarized against the building blocks of differentiated ART delivery for KPs put forth by the International AIDS Society [10]. Specifically, the Building Blocks “who,” “what,” “where,” and “when” of DTP implementation for FSW living with HIV who are not virally suppressed in the Siyaphambili study are described (Fig. 1).

#### When to implement DTP

When to implement DTP for FSW in Durban emerged as a relevant consideration as it determined whether women were at the venues and able to visit the van when it arrived. To optimize DTP uptake among FSW at greatest need, participants emphasized that DTP should be delivered during less busy work days as well as less busy work times; FSW recommended DTP during midweek, as Thursday through Sunday were busy days and Mondays were days of rest. Avoiding delivery on weekends and during pay periods or holidays due to high client demands was also emphasized by participants. FSW explained that a lack of consideration of when DTP strategies were delivered would result in hostility from some FSW (e.g. for interrupting work). Many participants suggested ART should be delivered in the morning, albeit not too early as some FSW work during the night. A few participants and many KIs expressed safety considerations for FSW and staff around delivery of DTP in the community late at night. A couple of home-based brothels were noted as having curfew for FSW and all services would need to be provided before then. Participants reported that considerations of when DTP is implemented would maximize uptake and acceptability.

#### Where to implement DTP

FSW noted that DTP should be delivered close to sex work venues, but away from public areas, shops, homes, and informal settlements. FSW expressed wanting to avoid being seen entering the mobile van by clients, boyfriends, or others who many assume they are sick or gossip about them for attending the mobile clinic. FSW recommended side streets and open spaces with minimal foot traffic as preferred DTP locations. A couple of women expressed disregard for others’ opinions, specifically noting that they would visit the mobile van wherever it parked as their health took priority. A few indoor-based FSW suggested parking the mobile van outside sex work venues and having staff deliver ART directly to the women’s rooms.

Safety around delivery locations also emerged, both in relation to staff safety on the mobile van as well as safe storage of ART once obtained by FSW. Record keeping of ART, operating during daylight hours, ensuring appropriate security measures of the mobile van (i.e. security personnel, male program staff), and having awareness and contingency plans while in the community were suggestions reported for ensuring staff and clinic safety. Participants also reported that the safety of receiving ART at sex work venues and appropriately storing ART would be dependent on where the woman stayed (i.e. in a shared vs. individual room), location of the place of residence (i.e. whether she sleeps at the venue where she works), and whether she had disclosed to peer or boyfriend cohabitants.

#### Whom to implement DTP

The provider of ART emerged as another important consideration to DTP implementation. FSW explained that staff who were not sensitized around sex work and operated at HIV clinics were barriers to ART access and adherence, and the implementation of DTP by sensitized staff could aid in overcoming these challenges. Participants noted that DTP should be implemented by sensitized nurses and peer educators. While some KIs suggested ART should either be delivered by foot, an unmarked van, or small vehicle to avoid HIV disclosure, these suggestions were not mentioned by FSW.

Branding on the mobile van and staff uniforms were expressed as an important consideration for DTP implementation as it had the potential for HIV and/or sex work disclosure. Several participants reported that branding the mobile van with ‘HIV,’ ‘sex workers,’ or ‘clinic’ would lead to stigma and discrimination from their pimps, boyfriends, or community. However, other FSW recommended maintaining existing mobile van branding, which includes 'HIV’ and ‘clinic’, to ensure recognition, authenticity, and security. Regarding nurse and peer uniforms, participants expressed professionality, with an emphasis on non-descript civilian attire and name tags, but no organizational branding. While some FSW noted that visiting a provider wearing a uniform identified them as sex workers, some KIs found uniforms to build trust and facilitate community-based activities.

#### What to implement with DTP

FSW recommended that ART packaging should be unidentifiable and dispensed with material to put in containers (e.g. cotton wool, sponge) to reduce recognizable sounds of pill movement. Participants reported considerations for ART storage, noting the need to ensure temperature control inside the mobile van. Most FSW expressed that the mobile van should offer HIV testing and treatment, but also sexually transmitted infections and tuberculosis screening, yeast infection medication, pap smears, and family planning. Additionally, almost all FSW expressed interest in psychosocial support as part of their HIV management. Psychological support was noted to relieve and manage stress and aid with disclosure, encouragement, parenting skills, and ART adherence support. Participants who expressed a desire for psychosocial support had histories of physical or sexual abuse and reported lacking adequate support systems.

## Discussion

Concerted efforts are needed to reach and sustain the most marginalized FSW living with HIV in South Africa with ART, to increase their quality and quantity of life and minimize onward transmission risks. These qualitative data highlight FSW-centered perspectives surrounding the opportunities and considerations for DTP implementation to support the appropriateness, adoption, and acceptability of the intervention. Additionally, these analyses aid in the understanding of for whom and in what context DTP implementation may be most cost-effective to retain and promote viral suppression among FSW in Durban, South Africa.

Several of the opportunities and considerations for DTP among FSW that emerged, including occupational influences, substance use, and mobility, were facilitators and barriers to ART care more generally and important considerations for the implementation of decentralized care. DTP was seen as an acceptable way to address these facilitators and barriers and determining strategies to optimally initiate and dispense DTP requires extensive consideration. Working within formal establishments (e.g. hotels, bars), HIV non-disclosure to non-paying partners, substance use, and mobility have been found to be associated with ART interruptions and barriers to retention in care and viral suppression among FSW [27, 44–49]. Safe venue-based ART delivery and storage while at work would depend on work location, housing conditions, and HIV disclosure, and recognizing venue typology becomes important for successful DTP uptake and implementation. Persons are more likely to miss a treatment dose or have suboptimal adherence if they have not disclosed their HIV status to their partners, co-workers or other peers [50, 51]. However, research among virally suppressed ART patients also shows that community ART distribution groups may be self-protective, normalizing and hiding their HIV status and reducing exposure to discrimination through fewer clinic visits [52]. The ability of DTP to serve as a safety net for women who are mentally incapacitated emerged but may pose challenges to DTP implementation due to FSW sobriety to engage in care, including bloodwork and counseling/treatment of other health ailments when found on site. Moreover, utility of DTP to provide onsite ART distribution may be an opportunity to retain FSW in care as women may move between clinic catchment areas, although potential implementation challenges may arise around scheduling DTP visits and meeting delivery targets given frequent mobility across venues.

The data support the understanding of for whom and in what context to implement DTP. FSW with economic barriers to clinic attendance and those that are highly mobile or recently relocated may benefit substantially from community-based DTP. Recognizing the heterogeneity in treatment needs among FSW, and tailoring DTP programs to those in greatest need may prove to be most cost-effective [35]. With financial limitations, including transportation costs and wages lost while at work, have been noted as drivers of treatment interruption [51, 53], the opportunity for FSW to save time and money with DTP at sex work venues emerged from the perspective of the user. Moreover, community-based DTP must balance preferences of FSW with implementation feasibility while maximizing reach and targeting those at highest need. FSW and KIs emphasized DTP should occur on less busy days and avoid peak hours, yet ART deliveries will only be effective when women are on site, which may not be the slowest times of the day or week. Implementation teams may frequent a sex work venue while unable to find patients, and implementation costs incurred with non-deliveries must be considered. Similarly, while night deliveries were not recommended, alternative strategies for how to reach nighttime workers are required.

Decentralized services are often implemented as “lighter touch” models, and the utilization of community and peers in DSD strategies engages FSW and removes the burden on the healthcare system through these lay providers [17]. However, from the FSW perspective, integrating other health services (e.g. reproductive health and non-communicable disease services) and psychosocial services into DTP emerged as important as it would allow for the dynamic needs and comorbidities of FSW to be addressed. Moreover, in consideration for whom and in what context to implement DTP, this more comprehensive approach may be particularly warranted for those who are not virally suppressed or most marginalized. Yet, the necessary intensity and feasibility of comprehensive service provision remains unclear. The current standard of care for most ART DSD programs in South Africa is to exclude patients with non-communicable diseases, such as hypertension and diabetes, or women who are currently pregnant [54]. DSD among key populations in other settings has been found as effective as facility-based care [31]. Recent implementation evaluation research surrounding the CCMDD program for stable adults living with HIV in South Africa found it to reduce stigma, but cited patient-level and organizational-level barriers (i.e. inadequate education about CCMDD, inability to access treatment on designated dates, challenges with communication and transportation, treatment packaging, rigidity of CCMDD rules, and insufficient infrastructure) [30]. Additional research is needed to assess whether these barriers and challenges exist in DSD implementation strategies aimed to support the unique needs of FSW living with HIV and what mechanisms and contextual considerations are needed to overcome DSD implementation among populations at increased risk for suboptimal HIV treatment outcomes. Furthermore, though there are indications that more comprehensive services are needed [55], FSW programs like THC in Durban, may have limited scope to offer integrated health services as outlined in national guidelines [56].

This study has several limitations. FSW participants were recruited from sex work venues served by THC in Durban, as well as THC peers. Though many of the themes raised are likely applicable in other regional settings, acceptability, and preferences may differ, particularly in areas in which programmatic services and trusting relationships have not already been developed. Through the community-based, peer-led recruitment process, FSW operating on virtual platforms (i.e. the websites or mobile applications) were not represented. However, FSW operating exclusively online are also unlikely to benefit or be reached by DTP in a sustainable manner. Maximum variation sampling ensured representation of FSW engaged and not engaged in care and operating across various sex work venues. Finally, themes that could have been explored further included contingency planning for DTP implementation, specifically focusing on what to do when FSW are not at the venue.

## Conclusions

This formative, qualitative analysis explored patient-centered opportunities and consideration for DTP implementation for FSW operating in Durban, South Africa. Considering FSW and KI perspectives is important when considering for whom and in what context to implement DTP as well as when identifying appropriate, acceptable, and feasible implementation strategies. Recognizing the heterogeneity in risks and needs among FSW is critical for cost-effective implementation, and these data suggest that tailoring DTP to preferences may promote ART adherence as well as address structural barriers to engagement and retention in care. Tensions between preferences and implementation considerations including feasibility and sustainability exist, and data are needed to determine the impact and cost-effectiveness of DTP in isolation or as part of a larger DSD package. Ultimately differentiated service delivery models, including DTP, may offer solutions to better addressing the treatment needs among FSW in South Africa, but effectiveness cannot be assumed, and evidence is urgently needed.

## Data Availability

The data generated and analyzed are not publicly available as these data are qualitative and within qualitative transcripts there are often ultimately identifiable pieces of information provided by the respondents. Further this is a criminalized population in South Africa. Thus, to protect respondents’ privacy and confidentiality the data are not publicly available. Data can be made available upon reasonable request and approval from both PIs Stefan Baral and Harry Hausler. Requesting individuals will need to be added to the study IRB prior to receiving the data.
